# nextflu: real-time tracking of seasonal influenza virus evolution in humans

**DOI:** 10.1093/bioinformatics/btv381

**Published:** 2015-06-26

**Authors:** Richard A. Neher, Trevor Bedford

**Affiliations:** ^1^Max Planck Institute for Developmental Biology, 72076 Tübingen, Germany and; ^2^Vaccine and Infectious Disease Division, Fred Hutchinson Cancer Research Center, Seattle, WA 98109, USA

## Abstract

**Summary:** Seasonal influenza viruses evolve rapidly, allowing them to evade immunity in their human hosts and reinfect previously infected individuals. Similarly, vaccines against seasonal influenza need to be updated frequently to protect against an evolving virus population. We have thus developed a processing pipeline and browser-based visualization that allows convenient exploration and analysis of the most recent influenza virus sequence data. This web-application displays a phylogenetic tree that can be decorated with additional information such as the viral genotype at specific sites, sampling location and derived statistics that have been shown to be predictive of future virus dynamics. In addition, mutation, genotype and clade frequency trajectories are calculated and displayed.

**Availability and implementation:** Python and Javascript source code is freely available from https://github.com/blab/nextflu, while the web-application is live at http://nextflu.org.

**Contact:** tbedford@fredhutch.org

## 1. Introduction

Every year, seasonal influenza infects between 10 and 20% of the global population, resulting in substantial human morbidity and mortality (World Health Organization, 2009). Vaccination remains the most effective public health measure to combat seasonal epidemics. However, influenza viruses constantly evolve and thereby undergo antigenic drift, allowing drifted viruses to reinfect individuals with acquired immunity to previously circulating strains. Owing to antigenic drift, the seasonal influenza vaccine needs frequent updating to remain effective. In any given year, the particular choice of vaccine strain plays a major role in determining vaccine efficacy and so it is of critical importance to develop tools to analyze the ongoing evolution of the influenza virus population in order to aid vaccine strain selection. The program **nextflu** presents a near real-time display of genetic relationships among influenza viruses and allows investigation of currently available sequence data. By visualizing many different genetic and epidemiological features, we hope that **nextflu** will help vaccine strain selection. Currently, **nextflu** tracks all four circulating lineages of seasonal influenza: A/H3N2, A/H1N1pdm, B/Victoria and B/Yamagata.

In implementation, **nextflu** consists of a processing pipeline written in Python called **augur** that analyzes virus sequence data and a JavaScript-based browser visualization called **auspice** that displays this processed information. As input, **augur** requires a FASTA file of sequences with header labels containing relevant information such as strain name, sampling date and passage history. For this purpose, influenza sequence data for the hemagglutinin (HA) gene is downloaded from the GISAID EpiFlu database (Bogner *et al.*, 2006), which contains the most up-to-date collection of seasonal influenza viruses. The first step in the processing pipeline is to automatically select a subset of representative viruses. Here, viruses without complete date or geographic information, viruses passaged in eggs and sequences <987 bases are removed. In addition, local outbreaks are filtered by keeping only one instance of identical sequences sampled at the same location on the same day. Following filtering, viruses are subsampled to achieve a more equitable temporal and geographic distribution. For our standard display period of 3 years and 32 viruses per month, this typically results in ∼1200 viruses, for which we align full-length HA sequences where available and partial sequences otherwise, using MAFFT (Katoh and Standley, 2013). Once aligned, the set of virus sequences is further cleaned by removing insertions relative to the outgroup to enforce canonical HA site numbering, by removing sequences that show either too much or too little divergence relative to the expectation given sampling date, and by removing known reassortant clusters, such as the triple-reassortant swine influenza viruses that have sporadically circulating since 2009 (Bastien *et al.*, 2010). As outgroup for each viral lineage, we chose a well characterized virus without insertions relative to the canonical amino-acid numbering and a sampling date a few years before the time interval of interest.

From the filtered and cleaned alignment, **augur** builds a phylogenetic tree using FastTree (Price *et al.*, 2009), which is then further refined using RAxML (Stamatakis, 2014). Next, the state of every internal node of the tree is inferred using a marginal maximum likelihood method and missing sequence data at phylogeny tips is filled with the nearest ancestral sequence at these sites. Internal branches without mutations are collapsed into polytomies. The final tree is decorated with the attributes to be displayed in the browser.

In addition to the phylogenetic tree, **augur** estimates the frequency trajectories of mutations, genotypes and clades in the tree. Frequencies are determined by maximizing the likelihood of sampling the observed set of virus sequences. In addition, we impose a smoothing that penalized rapid changes in frequency of the frequency derivative. **augur** estimates frequency with up to 1-month resolution. The result is similar to ‘allele dynamics’ plots in Steinbrück and McHardy (2011), but provides frequencies of clades in the tree in addition to point mutations. The **augur** pipeline is run every 3–7 days in response to sequence updates in the GISAID database.

**Fig. 1.**
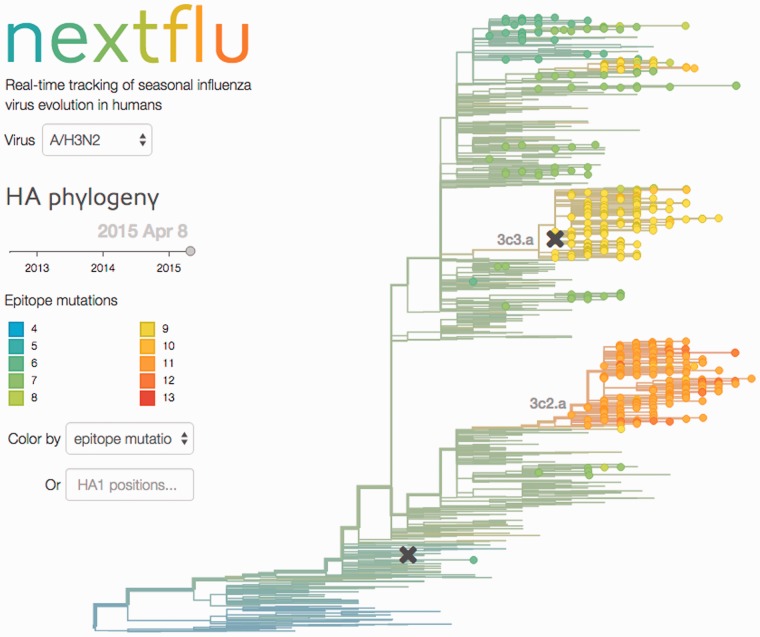
The **nextflu** website with the user interface on the left and the phylogenetic tree on the right

At the end of the **augur** pipeline, JSON files are exported containing the annotated phylogenetic tree, sequence data and frequency trajectories. These JSON files are then visualized by **auspice** using D^3^ (Bostock *et al.*, 2011) and a phylogenetic tree is displayed with branches scaled according to evolutionary distance across all sites (Fig. 1). The user can explore the data interactively by selecting viruses from different dates or by coloring the tree by attributes such as:

- *epitope mutations* at sites generally associated with antibody binding that have been suggested to be predictive of future clade success (Łuksza and Lässig, 2014),
- *receptor binding mutations* at seven positions close to the receptor binding site that have been shown to be responsible for major antigenic transitions in the past decades (Koel *et al.*, 2013),
- *local branching index* indicating the exponentially weighted tree length surrounding a node, which is associated with rapid branching and expansion of clades (Neher *et al.*, 2014),
- *HA genotype*, which directly colors the tree by genotype at specific amino acid positions.

The display can also be restricted to different geographic regions.

**Fig. 2.**
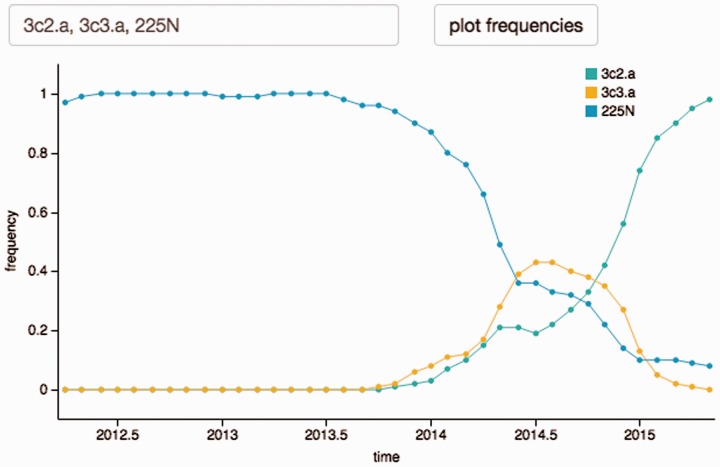
The frequency diagram allows geography-specific plotting of frequencies of individual mutations, pairs of mutations and clades in the tree

The frequency plot below the tree (Fig. 2) displays the frequency trajectory of clades in the tree whenever the mouse hovers above the branch defining the clade. Furthermore, trajectories of individual mutations, combinations of two mutations and predefined clades such as 3c3.a can be plotted. A second plot shows the variability of the alignment. On mouse-click on a variable position in this plot, **auspice** will color the tree by amino-acid at this position and plot its mutation frequencies.

We built **nextflu** to facilitate the analysis and exploration of seasonal influenza sequence data collected by laboratories around the world. By using the most recent data and integrating phylogenies with frequency trajectories and predictors of successful clades, we hope that **nextflu** can inform the choice of strains used in seasonal influenza vaccines. **nextflu** was designed to be readily adapted to other rapidly evolving viruses and we see significant room for future developments in this area.

## Funding

This work was supported by the ERC though Stg-260686 and by the NIH through U54 GM111274.

*Conflict of Interest*: none declared.
